# Adherence to ECG Monitoring Before Initiating Antipsychotics in Psychiatric Inpatients at Sultan Qaboos University Hospital: A Clinical Audit

**DOI:** 10.7759/cureus.107862

**Published:** 2026-04-28

**Authors:** Ahmed Al Harrasi, Zakya Al Difai

**Affiliations:** 1 Psychiatry and Behavioral Sciences, Oman Medical Specialty Board, Muscat, OMN; 2 Psychiatry, Sultan Qaboos University Hospital, Muscat, OMN

**Keywords:** antipsychotic agents, electrocardiography, hospital, inpatients, psychiatric department

## Abstract

Background: Antipsychotic medications are associated with QT interval prolongation and an increased risk of serious arrhythmias. Clinical guidelines, therefore, recommend baseline electrocardiogram (ECG) monitoring prior to initiation. This audit evaluated the compliance of baseline ECG monitoring before the initiation of antipsychotic medications in psychiatric inpatients at Sultan Qaboos University Hospital (SQUH) and explored the association of demographic and clinical factors.

Methods: A retrospective review was conducted of all psychiatric inpatients who were started on antipsychotics from January to December 2023. The records were checked to see if the ECGs were documented before the treatment. Descriptive statistical analysis, chi-square test, and t-test were applied to the data of age, sex, medical comorbidities, and prescribed antipsychotics.

Results: Out of 90 patients included in the analysis, 43 (47.8%) had a baseline ECG documented, and 47 (52.2%) had none. Patients of older age were more likely to have ECG monitoring performed (mean age 33.7 vs. 28.3 years, p = 0.090), but it was not statistically significant. There were no significant differences between gender, type of antipsychotic, or medical comorbidities.

Conclusion: These findings demonstrate that ECG monitoring before the initiation of antipsychotic medications at SQUH was found to be less than optimal. To enhance patient safety, standardized protocols, clinician education, and follow-up audits are needed.

## Introduction

Antipsychotic medications can lead to a lengthened QT interval, which may further increase the risk of development of serious arrhythmias, torsade de pointes (TdP), sudden death, etc. [1,2]. Antipsychotic-induced QT prolongation is primarily mediated through blockade of cardiac potassium channels (hERG), which delays cardiac repolarization and increases the risk of ventricular arrhythmias such as TdP [1,2]. Antipsychotics are broadly classified into first-generation (typical) and second-generation (atypical) agents, with varying risk of QT prolongation across individual drugs. Epidemiological studies have shown that antipsychotic use is associated with an increased risk of sudden cardiac death, necessitating baseline electrocardiogram (ECG) monitoring to detect QT interval abnormalities [3,4].

According to guidelines set by the National Institute for Health and Care Excellence (NICE) Clinical Guideline 178, the Maudsley Prescribing Guidelines, and the Royal College of Psychiatrists, baseline ECG monitoring is recommended prior to antipsychotic initiation to identify pre-existing QT prolongation and reduce the risk of drug-induced arrhythmias, particularly in inpatient settings or in patients at higher risk [5-7]. Nonetheless, audits worldwide show incomplete compliance with these recommendations, with adherence ranging from 23.3% to more than 90% depending on the setting [8-11].

However, to our knowledge, there are no published audits or study data from Oman evaluating adherence to ECG monitoring before antipsychotic initiation, leaving a gap in understanding local practice compliance with international standards. This audit assesses ECG monitoring compliance pre-antipsychotic initiation at Sultan Qaboos University Hospital (SQUH). It highlights areas for potential quality improvement.

## Materials and methods

The audit had a retrospective design involving the psychiatric inpatient unit of SQUH over one year, from January to December 2023. The study population included all consecutive psychiatric inpatients (both adults and children) who were started on any antipsychotic medications for the first time during their inpatient stay. Patients who were already receiving antipsychotic medication before admission were excluded.

The primary outcome was compliance with the recommended practices for ECG monitoring prior to antipsychotic initiation. Electronic patient records (EPR) were reviewed to determine whether a baseline ECG was performed before the first dose of antipsychotic medication. More data were collected to investigate possible demographic or clinical factors related to adherence. These were age, sex, comorbidity, and specific antipsychotic.

All data was collected using a standardized extraction sheet. Descriptive statistics, chi-square for categorical variables, and an independent samples t-test were used for continuous variables. Descriptive statistics summarized adherence rates and patient characteristics. Chi-square tests were used to see the association of categorical variables (e.g., gender and type of medication). Independent t-tests were used to assess the difference in mean age between patients with and without an ECG, assuming an approximately normal distribution of the data, given the sample size. A p-value of <0.05 was considered statistically significant. Statistical analysis was performed using Microsoft Excel (Microsoft Corporation, Redmond, Washington, United States). The referenced international clinical guidelines included NICE Clinical Guideline 178, the Maudsley Prescribing Guidelines, and the Royal College of Psychiatrists. They stated that psychiatric inpatients starting on antipsychotics should have an ECG performed as a baseline [5-7].

Ethics statement

This study was conducted as a retrospective clinical audit. In accordance with institutional policy, formal Institutional Review Board (IRB) approval was not required; therefore, no IRB approval number was issued. Approval to conduct the audit was obtained through the hospital audit process, and all data were anonymized.

## Results

The audit sample included 90 psychiatric inpatients who were initiated on antipsychotics. Out of these, 43 patients (47.8%) had documentation regarding the performance of an ECG before initiating the antipsychotic. Out of the total patients, 47 patients (52.2%) had no evidence of ECG monitoring before treatment, thereby showing a lack of compliance with the guidelines.

The participant characteristics were similar in terms of sex, with 47 males (52.2%) and 43 females (47.8%). The difference in ECG adherence by gender was not statistically significant (males, 55.8% vs. females, 44.2%) (χ² = 0.19, p = 0.659). Patients who had a baseline ECG were older (mean 33.7 years, SD = 16.4) compared to those who did not have a baseline ECG (mean 28.3 years, SD = 13.1). While this difference did not reach the conventional threshold for significance (independent samples t-test, t = 1.72, p = 0.090), the observed difference of more than five years suggests that older patients may be more likely to have baseline ECG monitoring, which may be clinically relevant.

The analysis of the antipsychotic drugs had no statistically significant difference related to ECG monitoring in different types of drugs (χ² = 3.70, p = 0.296). Risperidone, olanzapine, haloperidol, and quetiapine were commonly prescribed agents. Patients with medical comorbidities showed no significant association with ECG monitoring (χ² = 1.88, p = 0.391), though these patients with documented comorbidities were slightly more represented in the ECG group. A detailed summary of the findings is presented in Table 1.

**Table 1:** Comparison of patient characteristics and ECG monitoring status (n = 90). Data are presented as mean ± standard deviation (SD) for continuous variables and number (N) with percentage (%) for categorical variables. Independent samples t-tests (t) were used to compare continuous variables, and chi-square tests (χ²) were used to examine associations between categorical variables. A p-value <0.05 was considered statistically significant.

Variable	ECG Done (n = 43)	ECG Not Done (n = 47)	Test statistic, p-value
Mean Age (years, SD)	33.7 (16.4)	28.3 (13.1)	t = 1.72, p = 0.090
Gender (%)			χ² = 0.19, p = 0.659
Male	24 (55.8%)	23 (48.9%)	
Female	19 (44.2%)	24 (51.1%)	
Common Antipsychotics (%)			χ² = 3.70, p = 0.296
Olanzapine	15 (34.9%)	20 (42.6%)	
Risperidone	20 (46.5%)	13 (27.7%)	
Haloperidol	6 (14.0%)	10 (21.3%)	
Quetiapine	2 (4.7%)	4 (8.5%)	
Medical Comorbidities (%)			χ² = 1.88, p = 0.391
Present	11 (25.6%)	8 (17.0%)	
Absent	20 (46.5%)	20 (42.6%)	
Unknown	12 (27.9%)	19 (40.4%)	

## Discussion

Fewer than half of psychiatric inpatients had an ECG done before antipsychotic initiation, indicative of poor compliance. Although this finding is higher than that of some international studies (e.g., Hong Kong at 23.3%) [8], the rate remains lower than recommended by NICE, the Maudsley Prescribing Guidelines, and the Royal College of Psychiatrists [5-7]. This is particularly relevant given that commonly used antipsychotics such as haloperidol and quetiapine are associated with moderate QT prolongation, while risperidone and olanzapine have relatively lower effects [6].

Older patients were more likely to have a baseline ECG, perhaps through clinical awareness of increased cardiac risk, which has been observed by others [12]. Previous studies have shown that QT interval duration increases with age and that older age, female sex, and medical comorbidities are associated with a higher prevalence of QTc prolongation in patients receiving antipsychotics [13,14]. Even though in our audit the difference between the mean age of the groups was not statistically significant, this trend may have clinical relevance. No significant correlations were found between ECG adherence and gender, drug type, or the presence of medical comorbidities. Furthermore, the absence of heightened monitoring in patients with physical comorbidities, despite their higher susceptibility to cardiac complications, signifies missed chances for risk-based screening. The similar application of the ECG across different antipsychotic agents suggests that ECG monitoring was not done based on drug risk profiles. ECG monitoring primarily identifies QTc prolongation, which may necessitate dose adjustment, switching to lower-risk antipsychotics, or cardiology evaluation to reduce the risk of arrhythmias [5-7].

While this audit did not investigate the reasons for poor adherence, similar studies have reported documentation gaps, limited staff awareness, and absent standard operating procedures as potential reasons [10].

A key strength of this audit is that, to our knowledge, it represents the first evaluation of ECG monitoring adherence in a psychiatric inpatient setting in Oman. By providing a local benchmark against international guidelines, it highlights an important quality-of-care gap and offers a foundation for future quality improvement initiatives.

This audit has limitations, including being single-centered, retrospective, and based on a relatively small sample, which may limit the generalizability of the findings to other settings. In addition, incomplete documentation and a lack of detailed categorization of comorbidities, including potential cardiac conditions, may have affected subgroup comparisons.

Despite these limitations, the findings underline the need for targeted interventions, including standardized ECG monitoring protocols, improved staff training, and follow-up audits to ensure sustained adherence to guideline-based practice.

## Conclusions

The low adherence to ECG monitoring before starting antipsychotics at SQUH shows that patient safety can be improved in this area. To improve adherence and achieve more favorable patient outcomes, it is vital to implement standardized monitoring protocols as well as clinicians and staff education. Follow-up audits are recommended to ensure sustained adherence to guideline-based practice.
